# Supplementary material for: Gross domestic product and logistics performance index drive the world trade: A study based on all continents
Source: PLoS One. 2022 Mar 3;17(3):e0264474. doi: 10.1371/journal.pone.0264474 (PMC8893649; doi:10.1371/journal.pone.0264474)
Supplement: Attachment — Submitted filename: Response to Reviewer 1.docx [file pone.0264474.s002.docx]

**Point–by–point response to reviewer 1**

Previous submission was considered only the Asian and European Countries situation. However, considering the previous comments entire paper was redeveloped and covered the remaining continents as well. This study was the first of its kind to explore the impact of gross domestic product and logistics performance index on international trade of nations across worldwide.

| **Comments of Reviewer 1** | **Comments of Authors** |
| --- | --- |
| 1. The introduction section needs to focus more on the background of the study rather than defining terms including logistic performance index (LPI), and GDP. For example, the authors should discuss why this is study is important, i.e., if there is any gap in existing literature, or any debate, and how this study contributes to that. | Comment has been noted and this has been corrected in the revised manuscript. Paper has been drastically changed. Previous submission (PONE-D-21-30445) was considered only the Global, Asian and European Countries. Now the current paper has considered the remaining continents as well. The paper has revised accordingly considering the previous comments. |
| 2. The statement, “Recent research has not shown the influence of the LPI on international commerce (exports and imports)” in the second last paragraph of page 4 is not true. For example, Tanga and Abosedra (2019), similar to Gani (2017), Chakraborty and Mukherjee (2016), and Puertas, Martí, and Gracía (2014), found that logistics performance plays a vital role in enhancing the contribution of exports to economic growth. This statement further contradicts the literature review sections provided by the authors. In that case, the authors need to identify their unique contributions to the literature. | Comment has been noted and this has been corrected in the revised manuscript.  . |
| 3. The author's name in reference (14) on the third line of page 6 is missing. | Thank you. Comment has been noted and this has been corrected in the revised manuscript. |
| 4. I did not find any link between figure 1 and discussed literature provided by the authors as claimed by the authors. | Thank you and comment has been noted. This has been corrected in the revised manuscript. |
| 5. My main concern about the whole paper is the specification of the model. The models are not correctly specified as in equations (1), and (2). First, there is a strong possibility of reverse causality between net exports, and GDP; and net exports and purchasing power parity. As we can see from the literature and theory that GDP is strongly affected by next exports, the authors may want to be very careful about their identification in the presence of reverse causality. One of the solutions for that could be the use of instrumental variables. Second, while it is not uncommon to use interaction terms in this literature, the authors did not provide any justification or reference for using these specific interaction terms in equation (2). | Comment is well noted. The purpose of this study is to examine the impact of GDP and LPI on international trade, which determines the country's NEX. Two static linear panel models are developed based on the conceptual framework and the literature review. These models were also well endorsed in past literature. For example, please see following paper.  Koh BH, Wong WP, Tang CF, Lim MK. The double-edge resource-based view of logistics performance and governance in Asian countries. Asia Pacific Journal of Marketing and Logistics. 2018;30(3):652-68. doi: 10.1108/APJML-07-2017-0135.  <https://www.emerald.com/insight/content/doi/10.1108/APJML-07-2017-0135/full/html> |
| 6. The authors need to check their data again. As can be seen from Table 1 on page 9, the minimum GDP is USD 450.55. This is very unrealistic. I suggest the authors check their data again and estimate the model. | Thank you very much. Previous submission, there was a data entering error for the GDP. Data was rechecked and corrected. Thank you for being pointed out this. |
| 7. The authors need to log transform their data. I understand they can not do it for the net exports as it has negative values, but for other variables, they should do it for better interpretability. | This was tested, however the final results are not going to changed. In addition, past literature also used these variables without log transform suggesting no specification error to examine the impact of GDP and LPI on international trade. Please see following article,  Koh BH, Wong WP, Tang CF, Lim MK. The double-edge resource-based view of logistics performance and governance in Asian countries. Asia Pacific Journal of Marketing and Logistics. 2018;30(3):652-68. doi: 10.1108/APJML-07-2017-0135.  <https://www.emerald.com/insight/content/doi/10.1108/APJML-07-2017-0135/full/html> |
| 8. There is no robustness check of this study. I suggest the authors conduct a GMM analysis that is very common in this literature as mentioned by Tanga and Abosedra (2019) | GMM is possible when we want to have the lagged dependent variable as our independent variable or when the dependent variable has high persistence or long memory. (If that is the case then, fixed and random effects estimate would be inconsistent). However, LPI can collect only for 2007, 2010, 2012, 2014, 2016, and 2018 year. Thus, using lag variable for inconsistent time periods can create serious interpretation error. Thus, authors would like to use the pane regression since it was well known for analyzing this type of problems. |
